# Phase I/II Clinical Trial of Autologous Activated Platelet-Rich Plasma (aaPRP) in the Treatment of Severe Coronavirus Disease 2019 (COVID-19) Patients

**DOI:** 10.1155/2021/5531873

**Published:** 2021-07-07

**Authors:** Karina Karina, Iis Rosliana, Imam Rosadi, Siti Sobariah, Louis Martin Christoffel, Rita Novariani, Siti Rosidah, Novy Fatkhurohman, Yuli Hertati, Nurlaela Puspitaningrum, Wismo Reja Subroto, Irsyah Afini, Difky Ernanda

**Affiliations:** ^1^HayandraLab, Yayasan Hayandra Peduli, Jakarta, Indonesia; ^2^Klinik Hayandra, Yayasan Hayandra Peduli, Jakarta, Indonesia; ^3^Universitas Pembangunan Nasional Veteran Jakarta, Depok, Indonesia; ^4^Pusat Kajian Stem Cell, Universitas Pembangunan Nasional Veteran Jakarta, Depok, Indonesia; ^5^Department of Biology, Faculty of Mathematics and Natural Sciences, Mulawarman University, Samarinda, Indonesia; ^6^Koja Regional Public Hospital, Jakarta, Indonesia

## Abstract

**Background:**

The outbreak of Coronavirus Disease 2019 (COVID-19) has been increasing rapidly. This disease causes an increase in proinflammatory cytokine production that leads to cytokine storm or cytokine release syndrome (CRS). Autologous activated platelet-rich plasma (aaPRP) contains various types of growth factors and anti-inflammatory cytokines that may have the potential to suppress CRS. This study of phase I/II trial was aimed to evaluate the safety and efficacy of aaPRP to treat severe COVID-19 patients.

**Methods:**

A total of 10 severe COVID-19 patients from Koja Regional Public Hospital (Koja RPH) were admitted to the intensive care unit (ICU). All patients received aaPRP administration three times. Primary outcomes involving the duration of hospitalization, oxygen needs, time of recovery, and mortality were observed. Secondary outcomes involving C-reactive protein (CRP), neutrophil, lymphocyte, and lymphocyte-to-CRP (LCR) and neutrophil-lymphocyte ratio (NLR) were analyzed.

**Results:**

All patients were transferred to the ICU with a median duration of 9 days. All patients received oxygen at enrollment and nine of ten patients recovered from the ICU and transferred to the ward room. There was one patient who passed away in the ICU due to heart failure. The results of secondary outcomes showed that CRP value and lymphocytes counts were significantly decreased while neutrophils, LCR, and NLR were slightly increased after aaPRP administration.

**Conclusions:**

Our results of the phase I/II trial demonstrated that the use of aaPRP in severe COVID-19 patients was safe and not associated with serious adverse events, which showed that aaPRP was a promising adjunctive therapy for severe COVID-19 patients.

## 1. Introduction

In early 2020, the outbreak of Severe Acute Respiratory Syndrome Coronavirus-2 (SARS-CoV-2) was confirmed. The disease was named Coronavirus Disease 2019 (COVID-19), causing a pneumonia-associated respiratory syndrome [1]. The virus is known to have originated in Wuhan, China. On the 2^nd^ of March 2020, the Indonesian government confirmed two patients who were infected by SARS-CoV-2 [2]. The number of cases then increased drastically and reached 194,109 cases with 8,025 fatal cases (4.1%), 138,575 cases of recovery (71.4%), and 47,509 cases under treatment (24.5%) on September 7, 2020 [2].

The symptoms of COVID-19 ranged from no symptoms to acute bilateral pneumonia requiring hospitalization. The symptoms include fever, fatigue, dry cough, with laboratory tests often showing lymphopenia and elevated levels of lactate dehydrogenase [1]. The SARS-CoV-2 virus interferes with the normal immune response, causing the immune system to be compromised; hence, the inflammatory response is not controlled to disease severity [3]. Lymphopenia, activation and dysfunction of lymphocyte, granulocyte, and monocyte abnormalities, and increased proinflammatory cytokine production are the characteristics of a cytokine storm or cytokine release syndrome (CRS) condition, which is common in severe COVID-19 patients [3].

The cytokine storm that occurs causes inflammatory injuries to the capillary-alveoli membrane, which increases the permeability of the lungs. There is also the exudation of protein-rich pulmonary edema fluid into the air sacs, which in turn causes acute respiratory distress syndrome (ARDS), multiple organ failure, and a large number of deaths in COVID-19 patients [1, 3]. Some nonspecific inflammatory markers such as C-reactive protein (CRP), erythrocyte sedimentation rate (ESR), ferritin, fibrinogen, and D-dimers were found to be elevated in COVID-19 [4]. On the third day after the onset of disease, leukocytes and neutrophils in patients with severe symptoms were also found to be elevated, while lymphocytes, monocytes, T cells, T cells with CD8+, T cells with CD4+, and B cells decreased when compared to COVID-19 patients with moderate symptoms [5].

In several studies, it was found that the interleukin 6 (IL-6) level was elevated in the COVID-19 patients' serum and this was related to the severity of the disease [5]. IL-6 has an important role to play in acute inflammatory. It is known that IL-6 production is increased in the presence of TNF*α* and IL-1*β* [6]. Tocilizumab, an inhibitor for the IL-6 receptor, has been used in COVID-19 patients with severe cytokine storms. The use of tocilizumab can suppress IL-6 levels and can reduce the symptoms of COVID-19 [7]. However, the treatment of COVID-19 with tocilizumab is expensive and requires hospitalization, and the secondary side effects are not yet clear [8]. Phase III clinical trials for tocilizumab in COVID-19 patients have not yet been completed to identify the long-term side effects of tocilizumab [8].

Another approach that was used to reduce cytokine storm in COVID-19 patients was mesenchymal stem cells (MSCs) therapy [9–11]. MSCs are known to secrete a wide variety of trophic factors, cytokines, and growth factors that have immunomodulating and anti-inflammatory effects. A study on 7 COVID-19 patients using MSCs: four of them had severe symptoms, one was critical, and two others had common symptoms, which showed the potential for MSCs in the management of COVID-19 [10]. As much as 1 × 10^6^ per kg body weight of MSCs was given intravenously when each patient's condition worsened. Symptoms (high fever, decreased oxygen saturation, dyspnea, and pneumonia) were subsided on the second to fourth day after administration of MSCs. Chest CT scan results showed a significantly reduced pneumonia infiltration. In fact, there was a significant clinical improvement in elderly patients who presented with critical conditions. In another study, the potential of MSCs in the management of COVID-19 was shown in COVID-19 patients who were critically ill and had to be assisted with a ventilator [11]. The patient received an infusion of 5 × 10^7^ MSCs from cord blood every 3 days, and the patient was able to walk again in 4 days. After the second infusion of MSCs, the T cell count returned to normal. To date, 17 clinical trials regarding the immunomodulating and anti-inflammatory potential of MSCs in the management of COVID-19 have been completed [9]. Unfortunately, therapy for MSCs is still quite expensive, as the production of MSCs can only be done in few laboratories that have received permission from the Ministry of Health of the Republic of Indonesia.

The most common MSCs therapy in Indonesia is autologous MSCs therapy. However, the autologous MSCs are not suitable for acute illnesses such as COVID-19 because MSCs need to be cultured for a period of at least 2-3 weeks to obtain a sufficient number of MSCs for clinical application. In contrast, platelet-rich plasma (PRP) can be obtained in a short time, easily with simple centrifugation, and required relatively low production costs. PRP contains various types of growth factors, such as transforming growth factor beta (TGF-*β*), platelet-derived growth factor (PDGF), vascular endothelial growth factor (VEGF), basic fibroblast growth factor (bFGF), insulin-like growth factor 1 (IGF-1), hepatocyte growth factor (HGF), and epidermal growth factor (EGF) [12, 13]. The growth factors released by platelets suppress the production of IL-6, TNF*α*, and IL-1*β* by synoviocytes. In the inflammatory phase, the TGF-*β* plays a role as an immunosuppressor and inhibits the expression of proinflammatory cytokines in autoimmune diseases, such as rheumatoid arthritis [14]. PRP also contains anti-inflammatory cytokines such as interleukin-1 receptor antagonist (IL-1RA) [15] that can inhibit the secretion of IL-6 in cancer cells [16].

Karina et al. [17] reported that the platelet count of PRP in diabetic patients was not statistically significant compared to healthy patients, whereas the concentration of vascular endothelial growth factor (VEGF) protein was significantly higher in diabetic patients than healthy patients [17]. It shows that their bodies regulate the production of protein or cytokine depending on their needs. We suggest that the platelet in COVID-19 patients can also produce anti-inflammatory cytokines to manage cytokine storms in their bodies, although the mechanism is still unclear. Based on the various results of these studies, PRP is suggested to have clinical potential that can be applied in the management of COVID-19.

The PRP that was developed for its safety in this study was the autologous activated PRP (aaPRP) and was administered intravenously. Intravenous use of aaPRP contained no platelets and leukocytes, which may induce adverse events. Thus, it showed that intravenous aaPRP produced no adverse events such as coagulation problems, infections, and allergic reactions. Furthermore, intravenous use in various pathological conditions, such as diabetes mellitus, hypertension, stroke, osteoarthritis, postcardiac stenting, antiaging, and various other pathologies, showed no adverse events and is proven to be safe [18]. The combination of aaPRP with a stromal vascular fraction (SVF) administered through infusion was also reported to have no serious side effects in 421 patients [19]. Thus, this study analyzes the potential of aaPRP as an intravenous treatment for severe COVID-19 cases.

## 2. Methods

### 2.1. Study Design and Patient

This study was an open-label study to investigate the safety and early efficacy of aaPRP infusion in severe COVID-19 patients. Ethical clearance was obtained from the Health Research Ethics Committee, University of Indonesia, and Cipto Mangunkusumo Hospital with letter of approval No. 1508/UN2.F1/ETIK/PPM.00.02/2020. This report was a preliminary study that is part of a clinical trial comparing the standard national protocol with Avigan and action with/without aaPRP. This study was registered to the clinicaltrials.gov with identifier NCT04715360. Ten patients were recruited in the Intensive Care Unit of Koja Regional Public Hospital (Koja RPH), Jakarta, Indonesia, with written informed consent. The subjects were 10 COVID-19 patients (probable/rapid reactive/confirmed).

The inclusion criteria were male and female, 18−65 years old with fever or symptoms of respiratory infection, respiratory rate > 30 times per minute, severe respiratory distress, oxygen saturation < 90% while breathing ambient air, and showing a pattern of ground glass-opacity pattern in the lung, with severe pneumonia to sepsis. The exclusion criteria were HIV-positive, hepatitis, pregnancy, destroyed lung, and cancer patients.

During the study, all patients received oxygen therapy and were given medications including dexamethasone 1 × 5 mg/IV, Avigan 2 × 1.600 mg for a day and 2 × 600 mg for five consecutive days, ceftriaxone injection 1 × 2 g/IV for five days and then switched to oral tablet cefixime 2 × 200 mg, and also subcutaneous heparin 1 × 5000 IU.

### 2.2. Autologous Activated Platelet-Rich Plasma (aaPRP)

The venous blood samples were collected aseptically using a winged needle to 8 citrate tubes of BD Vacutainer, 1.2% of buffered sodium citrate (each tube contains 3 mL of blood) [17, 18]. Blood for aaPRP is then centrifuged at 1000 rpm (188 × g) for 10 minutes (Thermo Scientific Sorvall ST8, USA) until the plasma and red blood cells were separated. The plasma was then carefully collected with a transfer pipette and transferred into two sterile 15 mL tubes. The plasma was centrifuged again at 3000 rpm (1690 × g) for 10 minutes (Thermo Scientific Sorvall ST8, USA). Then, the upper plasma (platelet-poor plasma, PPP) was aspirated until 3 mL of plasma was left in the tube followed by plasma homogenization. This remaining plasma is the PRP. Thus, the two tubes containing 3 mL of PRP then were combined into one tube, homogenized by pipetting up and down. The calcium activator (H-Remedy, Hayandra, Indonesia) solution was added to induce fibrin clots formation. The fibrin clot was removed to isolate the autologous activated PRP (aaPRP). The aaPRP was injected into a 100 mL 0.9% NaCl infusion bag.

### 2.3. aaPRP Administration

The aaPRP that was prepared in a 100 mL 0.9% NaCl infusion bag was then infused intravenously for 10–15 minutes with blood transfusion set. Each patient received aaPRP therapy for up to 3 times. Intravenous aaPRP was given on days 1, 3, and 5 after the patients was transferred to the ICU.

### 2.4. Data Collection

**Figure 1:**
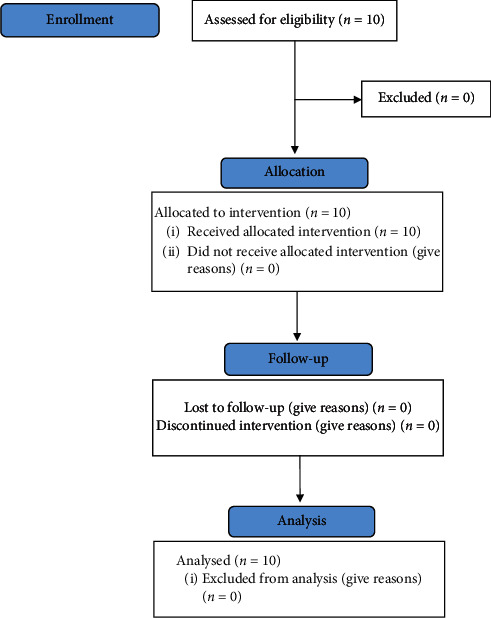
CONSORT flow diagram.

This research study acquired and evaluated demographic data, patient medical history, clinical examination results, medication during hospitalization period in ICU, C-reactive protein (CRP), neutrophil, lymphocyte, and lymphocyte-to-CRP (LCR) and neutrophil-lymphocyte ratio (NLR). Patients were monitored daily during their hospitalization. The primary outcome was the time of hospitalization, level of oxygen therapy, and their recovery or mortality, defined as the first day when patients met the criteria as inclusion criteria for aaPRP treatment. The secondary outcome was clinical laboratory data measured before and after during aaPRP treatment and hospitalization until the patients were discharged. The clinical laboratory data included CRP, neutrophil, lymphocyte, LCR, and NLR. Short-term side effects and complications that occurred after aaPRP administration and the number of patients who passed away in ICU were recorded for analysis. See Figure 1 for the CONSORT diagram.

### 2.5. Statistical Analysis

Data were presented in mean and standard deviation (SD). Numeric data were tested with the Shapiro–Wilk test for data distribution. A significant difference between pre- and post-aaPRP infusion for data with normal distribution were tested with paired *t*-test, while nonparametric test for related samples was tested with Wilcoxon test. A *p*-value below 0.05 was considered statistically significant.

## 3. Results

### 3.1. Platelet-Rich Plasma Characterization

The administered PRP was the activated PRP with calcium activator, where no platelet, no erythrocytes, and no leucocytes were observed. Basal platelet concentration in blood was 395 × 10^6^ platelets/*µ*L, while platelet concentration in PRP (nonactivated) was more than 1.20 × 10^9^ platelets/*µ*L. Thus, the PRP used in this study was 310-00-11 as per code formulated by [20], and the characteristics of PRP are reported in Table 1.

### 3.2. Patients

A total of 10 patients who had the severe COVID-19 condition were transferred to the intensive care unit (ICU), where they received aaPRP three times. The mean age of patients was 52.1 years, and 40% were male (Table 2). All patients had either two (40%) or three (40%) or four (20%) of the coexisting conditions at enrollment, most commonly DIC, CAP, type 2 diabetes, CHP, and hypertension (Table 2).

### 3.3. Primary Outcomes

Median duration of all patients from initial hospitalization in the emergency room to ward room and in the ICU was 4 days and 9 days consecutively, and the median duration from emergency room to ICU was 17 days. Due to worsening conditions, all patients received oxygen at enrollment (Table 3). There were three types of oxygen support, which were NRM (20%), HFNC (50%), and ventilator (30%). Median days of patients receiving oxygen using NRM, HFNC, and ventilator were 5, 9, and 6 days. Patients (90) who were given PRP were recovered after 9 days. However, there was one patient who passed away due to heart failure in the ICU.

### 3.4. Secondary Outcomes

After the safety data were monitored, the CRP, neutrophil, lymphocyte, LCR, and NLR were evaluated. The results showed that CRP level was significantly decreased (*p*=0.005) after aaPRP was administered to the patients compared to CRP level before aaPRP treatment (Table 4). Neutrophils (*p*=0.389) and NLR (*p*=0.285) counts were increased but were not statistically significant after aaPRP treatment. However, lymphocyte count was decreased (*p*=0.234) and LCR was significantly increased (*p*=0.009), which showed signs of inflammation reduction. These results indicated that there were positive correlations between decreased CRP level and lymphocyte counts and increased neutrophil and NLR value. This can be concluded that the patients may still have the infection but showed low inflammation levels.

Our results of the phase I/II trial demonstrated that the use of aaPRP in severe COVID-19 patients was safe and not associated with serious adverse results.

## 4. Discussion

The state of COVID-19 in Indonesia has reached a new level of urgency. Therefore, it is paramount to research a safe and effective therapy to manage COVID-19 symptoms. The existing regulations in Indonesia are limiting the use of allogenic cell therapy, which makes the research in this area require its own approval from the Indonesian FDA. Reflecting on our experience from over 4,200 cases with/without existing diseases of using aaPRP safely [18], we managed to start this clinical trial using aaPRP. In this study, we successfully administered aaPRP intravenously to lead bioactive molecules, including anti-inflammatory cytokines to pulmonary alveoli lung. The final product of our aaPRP was 6 mL diluted in 100 mL of NaCl 0.9%. No side effect was observed. Even though there are still possibilities of increasing the dosage for aaPRP, we applied the processing technique as we already administered, reported, and registered [18]. The association between dose and efficacy of aaPRP needs to be explored in further studies. Another possible PRP administration is via airway that would reach pulmonary alveoli lung directly [21]. The possibilities of PRP as a treatment against the respiratory condition were also reported by [21, 22].

Our current findings showed that hyperinflammation is a reliable indicator of COVID-19 severity and ICU admission [23]. Inflammation first occurred locally in the postprimer infection site. Infected cells then activated a large number of white blood cells, leading to the production of many inflammatory cytokines. Inflammatory signals recruited more white blood cells to migrate to the site of infection and activated them [24]. These inflammatory cytokines then spread systemically throughout the body, which can be seen on the high levels of systemic CRP in this study and other studies [5, 25]. The systemic levels of several proinflammatory cytokines such as IL-2, monocyte chemoattractant protein-1 (MCP-1), macrophage inflammatory protein-1A (MIP-1A), tumor necrosis factor-*α* (TNF*α*), and IL-6 were increased in ICU patients [26, 27]. In this condition, it was anticipated that the production of the anti-inflammatory cytokines would be suppressed.

Interestingly, the systemic level of IL-10 was increased in COVID-19 patients in the second week following symptom onset [28]. IL-10 is a potent immunomodulator with normal function, which can inhibit antigen-presenting cells, activation, and infiltration of macrophage into the site of injury, attenuating proinflammatory expression, and at the cellular level, promoting the specific destabilization of inflammatory cytokine mRNA by suppressing mRNA of stabilizing protein HuR (human antigen R) [29]. It is likely that immune systems try to control the hyperinflammation that is unfortunately too late. Furthermore, imbalance of pro- and anti-inflammatory systems in the initial phase of lung injury can result in failure to restore normal pulmonary architecture and lead to pulmonary fibrosis. Once fibrotic tissue was formed, pulmonary architectural distortion will be permanent, and lung dysfunction will be irreversible [30]. It also has been reported that long-term IL-10 exposure may actually promote fibrotic outcomes and exacerbate lung injury [30].

These findings had led us not only to find the medications that have good clinical safety and efficacy but also to deliver them at the right time. In this study, we found that giving aaPRP treatment as soon as the clinical condition was observed to be worsened may result in a shorter duration of hospitalization in ICU and decrease the need for ventilator support. CRP value after PRP treatment was lower than the initial value, indicating that clinical improvement was due to inflammation control. Although how aaPRP controls the inflammation remains unclear, aaPRP was known to contain many types of growth factors that control inflammation and stimulate the healing process [31]. VEGF is a potent angiogenic agent contained in aaPRP. VEGF, in the presence of TNF*α*, can interfere with the degradation of IkB that leads to reduced activation of NF-kB, a transcription factor that plays a pivotal role in inflammation and immunity [32]. It is known that NF-kB activation leads to the production of many proinflammatory cytokines, such as IL-1, IL-2, IL-6, IL-8, IL-12, and TNF*α* [33]. TNF*α* itself, once activated, can stimulate the activation of NF-kB that can exacerbate inflammation. Thus, suppression of NF-kB can control the inflammation, which may explain the biological function of aaPRP in COVID-19 patients. HGF, which is the main active component of the PRP, acts in intracellular pathways. It has an important role as an anti-inflammatory to suppress NF-kB and to reduce the cytokine storm in COVID-19 patients [21]. HGF also has antiapoptotic effect against proinflammatory stimuli towards epithelial cells [34], which is important as an angiogenic and a recovery agent for COVID-19 patients.

Inflammation is cross-linked to oxidative stress [35]. Antioxidants, such as zinc and n-acetylcysteine, are used in COVID-19 patients to reduce inflammation [36, 37]. Recent studies showed that several growth factors included in aaPRP, such as EGF [38] and IGF-1 [39], also have antioxidant effects. In addition, IGF-1 and hepatocyte growth factors were also known as antifibrogenic agents that help reduce inflammation-mediated fibrosis in the lungs of COVID-19 patients [40]. Furthermore, various growth factors in aaPRP can interact with endogenous stem and progenitor cells in lung tissue to start the repairing process that involves angiogenesis, fibroblast activation, and collagen deposition [41]. Angiogenic growth factor, such as VEGF and fibroblast growth factor, induces the migration and proliferation of uninjured endothelial cells leading to pulmonary capillary angiogenesis [42]. EGF and TGF-*α* stimulate the proliferation of endogenous stem cells for alveolar epithelium regeneration [43]. Fibroblastic invasion of the alveoli occurred and transformed into myofibroblast that leads to collagen deposition that restores pulmonary architectural distortion [41].

As expected, this study showed that the need for invasive mechanical ventilation decreased following administration of aaPRP. This indicates a potency of aaPRP treatment in promoting the acceleration of lung functioning recovery in COVID-19 patients from respiratory distress. A decreased need for invasive mechanical ventilator support was also found after the administration of IFN *β*-1b [44]. It also has been shown that the systemic level of IL-10 was increased markedly 40 hours after administration of single-dose IFN *β*-1b in multiple sclerosis patients [45]. It should be further investigated if aaPRP treatment will produce the same effect as administration of IFN *β*-1b and to explore other potential mechanisms of aaPRP in controlling cytokine storms as well. However, regarding safety, we do not observe any complications or serious adverse events, except those that were observed prior to aaPRP treatment. In other studies, administration of IFN *β*-1b frequently resulted in interferon-b-1b, which showed flu-like symptoms and injection-site reactions, lymphopenia, depression, suicidal ideation, and injection-site necrosis [46].

In the case of platelets alteration due to coronavirus infection, the pro- or anti-inflammatory level should be evaluated. Based on our outcomes, it seems PRP released anti-inflammatory cytokines to prevent cytokine storms. Our previous study of platelets and their protein concentration in diabetic donors compared to healthy donors showed that no significant difference in platelets number, but platelets from diabetic donors released a significantly higher amount of VEGF protein that plays a role in the angiogenic process [18]. In contrast, diabetic patients had a problem with their ability to heal the wound. It seems that platelets from diabetic donors cannot be activated (dysfunction) due to their microenvironment condition. Thus, this condition might also happen in platelets of COVID-19. However, the cytokine level of pro- and anti-inflammatory markers level is still unclear.

There has been a reference suggesting the use of PRP from healthy patients to be considered as an allogenic potential therapy for COVID-19 [21]. With the lack of our experiences of using allogenic PRP therapy, the comparison of autologous and allogenic PRP therapy for COVID-19 needs to be evaluated in further studies to prove the efficacy.

The results showed good clinical safety and efficacy of aaPRP treatment as cost-effective and feasible adjunctive therapy for severe COVID-19 patients. However, our study still had many limitations, including the small sample sizes and no evaluation of cytokines or growth factors related to pro- or anti-inflammatory. Thus, more studies should be conducted in a larger sample size to get the exact estimation of survival and clinical benefits of aaPRP treatment.

## 5. Conclusion

This study showed that aaPRP treatment is promising as adjunctive therapy for severe COVID-19 patients while waiting for the vaccine that will eradicate the viral infection. aaPRP treatment can be used on a compassionate basis, thanks to their potency in promoting inflammatory control, accelerating tissue regeneration, and improving clinical outcomes in severe COVID-19 patients. Multicenter studies with proper designs are needed to adequately establish the safety and efficacy of aaPRP in severe COVID-19 patients in Indonesia and worldwide. It is also beneficial to explore the benefit of aaPRP treatment for mild-to-moderate COVID-19 patients in order to reduce the need for ICU admissions.

## Figures and Tables

**Table 1:** Summary of autologous activated PRP characteristics

*1. PRP preparation*	

Initial blood volume	24 mL
Anticoagulant	Sodium citrate 1.2% (wt/V)
System	Open
Centrifugation	Yes
Number	2
Speed	1000 rpm (188 g), 3000 rpm (1690 g)
Final PRP volume	6 mL

*2. Autologous activated PRP characteristics*	

PRP type	310–00–11
Mean platelet volume (MPV)	—
Red blood cells	—
White blood cells	—
Neutrophils	—
Lymphocytes	—
Monocytes	—
Eosinophils	—
Basophils	—
Activation	Calcium activator

*3. Application characteristics*	

Formulation type	Liquid
Administration route	Intravenous (IV)
Dosage	Up to 3 dosages with a day of interval (day 1, 3, 5)
Volume	6 mL PRP + 100 mL 0.9% NaCl
Dose (number of injected platelets (range))	0 cell number

*4. Other remarkable PRP and study features*	

The final product of activated autologous PRP (contains no platelet) was administered (IV)	

**Table 2:** Demographic and clinical characteristics of the patients.

Characteristics	aaPRP treatment
*Characteristics*	
Age, yr	52.1 ± 8.89
Male sex, %	40
Female sex, %	60
No. of coexisting conditions, no./total no. (%)	
Two	4/10 (40)
Three	4/10 (40)
Four	2/10 (20)
Coexisting conditions, no./total no. (%)	
Type 2 diabetes	4/27 (14.81)
Hypertension	2/27 (7.41)
Congestive heart failure (CHF)	3/27 (11.11)
Disseminated intravascular coagulation (DIC)	7/27 (25.93)
Community-acquired pneumonia (CAP)	4/27 (14.81)
Coronary artery disease (CAD)	1/27 (3.70)
Chronic kidney disease (CKD)	1/27 (3.70)
Lung pulmonary tuberculosis	1/27 (3.70)
Atrial fibrillation	1/27 (3.70)
Hypo-Na	1/27 (3.70)
Metabolic encephalopathy	1/27 (3.70)
Happy hypoxia	1/27 (3.70)

**Table 3:** Primary outcomes of the patients.

Outcomes	aaPRP treatment
*Hospitalization*	
Median duration of all patients from initial hospitalization in emergency room to ward room, days	4 (1 to 8)
Median duration of all patients in ICU, days	9 (5 to 16)
Median duration of among patients from emergency room and ward room to ICU who recovered, days	17 (10 to 23)

*Oxygen*	
No. of patients/total no. (%)	10/10 (100)
Type of oxygen support, no./total no. (%)	
NRM	2/10 (20)
HFNC	5/10 (50)
Ventilator	3/10 (30)
Median days receiving oxygen–days	
NRM	5 (4 to 6)
HFNC	9 (7 to 14)
Ventilator	6 (3 to 6)

*Recovery*	
No. of recoveries, %	9/10 (90)
Median time to recovery, days	9 (5 to 16)

*Mortality*	
No of deaths, %	1/10 (10)
Median time to passed away, days	8

*Main cause of mortality*	
Heart failure, %	1/1 (100)

**Table 4:** Secondary outcomes based on CRP, neutrophil, lymphocyte, LCR, and NLR

No	CRP		Neutrophil		Lymphocyte		LCR		NLR	
	Before	After	Before	After	Before	After	Before	After	Before	After
1	17.30	0.94	91.30	88.80	4.30	6.60	0.25	7.02	21.23	13.45
2	22.58	1.51	85.30	88.30	9.30	4.50	0.41	2.98	9.17	19.62
3	8.57	0.95	72.50	88.10	18.30	4.30	2.14	4.53	3.96	20.49
4	9.48	1.17	90.20	82.20	4.70	10.50	0.50	8.97	19.19	7.83
5	8.99	0.56	94.00	85.20	2.60	7.70	0.29	13.75	36.15	11.06
6	7.74	6.44	82.30	81.20	8.50	10.20	1.10	1.58	9.68	7.96
7	5.79	1.98	70.60	76.80	20.60	13.90	3.56	7.02	3.43	5.53
8	4.48	0.63	73.70	80.10	18.90	12.80	4.22	20.32	3.90	6.26
9	23.00	6.10	82.60	78.80	11.90	12.80	0.52	2.10	6.94	6.16
10	5.06	5.03	71.70	92.60	17.10	4.40	3.38	0.87	4.19	21.05

Mean **±** SD	**10.89** **±** **8.09**	**2.53** **±** **2.36**	**81.42** **±** **8.82**	**84.21** **±** **5.15**	**19.37** **±** **22.95**	**8.77** **±** **3.76**	**1.64** **±** **1.55**	**6.91** **±** **6.15**	**11.79** **±** **10.66**	**11.94** **±** **6.30**

*p*	**0.005** ^*∗*^		0.389		0.234		**0.009** ^*∗*^		0.285	

## Data Availability

The data used to support the findings of this study are included within the article.

## Abbreviations

aaPRP: Autologous activated platelet-rich plasma
COVID-19: Coronavirus disease 2019
CRS: Cytokine release syndrome
LCR: Lymphocyte-to-CRP ratio
NLR: Neutrophil-lymphocyte ratio
CRP: C-reactive protein
DIC: Disseminated intravascular coagulation
CAP: Community-acquired pneumonia
CAD: Coronary artery disease
CKD: Chronic kidney disease
ARDS: Acute respiratory distress syndrome
ESR: Erythrocyte sedimentation rate
IL: Interleukin
MSCs: Mesenchymal stem cells
TGF-*β*: Transforming growth factor beta
PDGF: Platelet-derived growth factor
VEGF: Vascular endothelial growth factor
bFGF: Basic fibroblast growth factor
IGF-1: Insulin-like growth factor 1
HGF: Hepatocyte growth factor
EGF: Epidermal growth factor
IL-1RA: Interleukin-1 receptor antagonist
PPP: Platelet-poor plasma
ICU: Intensive care unit
MIP-1A: Macrophage inflammatory protein-1A
MCP-1: Monocyte chemoattractant protein-1
TNF*α*: Tumor necrosis factor-*α*.
